# *Francisella philomiragia* Infection and Lethality in Mammalian Tissue Culture Cell Models, *Galleria mellonella*, and BALB/c Mice

**DOI:** 10.3389/fmicb.2016.00696

**Published:** 2016-05-24

**Authors:** Crystal N. Propst, Stephanie L. Pylypko, Ryan J. Blower, Saira Ahmad, Mohammad Mansoor, Monique L. van Hoek

**Affiliations:** ^1^School of Systems Biology, George Mason University, Manassas, VAUSA; ^2^Department of Biology, George Mason University, Fairfax, VAUSA; ^3^National Center for Biodefense and Infectious Diseases, George Mason University, Manassas, VAUSA

**Keywords:** *Francisella philomiragia*, murine model, mammalian cells, *Galleria mellonella*, pulmonary infection, intranasal

## Abstract

*Francisella (F.) philomiragia* is a Gram-negative bacterium with a preference for brackish environments that has been implicated in causing bacterial infections in near-drowning victims. The purpose of this study was to characterize the ability of *F. philomiragia* to infect cultured mammalian cells, a commonly used invertebrate model, and, finally, to characterize the ability of *F. philomiragia* to infect BALB/c mice via the pulmonary (intranasal) route of infection. This study shows that *F. philomiragia* infects J774A.1 murine macrophage cells, HepG2 cells and A549 human Type II alveolar epithelial cells. However, replication rates vary depending on strain at 24 h. *F. philomiragia* infection after 24 h was found to be cytotoxic in human U937 macrophage-like cells and J774A.1 cells. This is in contrast to the findings that *F. philomiragia* was non-cytotoxic to human hepatocellular carcinoma cells, HepG2 cells and A549 cells. Differential cytotoxicity is a point for further study. Here, it was demonstrated that *F. philomiragia* grown in host-adapted conditions (BHI, pH 6.8) is sensitive to levofloxacin but shows increased resistance to the human cathelicidin LL-37 and murine cathelicidin mCRAMP when compared to related the *Francisella* species, *F. tularensis* subsp. *novicida* and *F. tularensis* subsp. *LVS*. Previous findings that LL-37 is strongly upregulated in A549 cells following *F. tularensis* subsp. *novicida* infection suggest that the level of antimicrobial peptide expression is not sufficient in cells to eradicate the intracellular bacteria. Finally, this study demonstrates that *F. philomiragia* is lethal in two *in vivo* models; *Galleria mellonella* via hemocoel injection, with a LD_50_ of 1.8 × 10^3^, and BALB/c mice by intranasal infection, with a LD_50_ of 3.45 × 10^3^. In conclusion, *F. philomiragia* may be a useful model organism to study the genus *Francisella*, particularly for those researchers with interest in studying microbial ecology or environmental strains of *Francisella*. Additionally, the Biosafety level 2 status of *F. philomiragia* makes it an attractive model for virulence and pathogenesis studies.

## Introduction

Members of the *Francisella* genus are small, non-motile, Gram-negative coccobacilli of the gamma-proteobacteria class (Sjostedt, 2007). *Francisella philomiragia* was first identified in an ailing muskrat located in Utah approximately 50 years ago, following the discovery of its related species, *F. tularensis* (Hollis et al., 1989). Mistakenly characterized as *Yersinia philomiragia* due to its 24% genomic homology with *Y. pestis* and similarities in morphology, it took 30 years to re-categorize the species as a member of the *Francisella* genus (Hollis et al., 1989).

*Francisella philomiragia* has an affinity for aquatic environments which may increase its host species potential (Anda et al., 2001; Tarnvik et al., 2004). The natural range of *F. philomiragia* reflects its preference for aquatic environments as it is found near bodies of water, particularly brackish or salt water in the mainland United States (Hollis et al., 1989; Whipp et al., 2003; Berrada and Telford, 2010; Siddaramappa et al., 2012; Whitehouse et al., 2012). *F. philomiragia* may exist naturally by forming biofilms on exposed surfaces of the environment and infecting the aquatic amoeba, *Acanthamoeba castellanii* (Verhoeven et al., 2010).

Virulence factors in *F. philomiragia* have not been well studied in this species, but likely include proteins encoded by the Francisella Pathogenicity Island (FPI) and phospholipase C (Zeytun et al., 2012), similar to other members of the *Francisella* genus (Nano and Schmerk, 2007; Dai et al., 2010).

A related species, *F. noatunensis* (formerly named *F. philomiragia noatunensis)*, is pathogenic to many fish and mollusk species, which inflicts negative economic and health effects on fisheries (Kay et al., 2006; Ostland et al., 2006; Mauel et al., 2007; Mikalsen and Colquhoun, 2009). Mikalsen et al. (2009) previously asserted that *F. philomiragia* subsp. *noatunensis* is a fish pathogen that is not lethal to mice and does not pose a threat to human health (Mikalsen et al., 2009). However, in the time since that publication, *F. noatunensis* has been elevated to species level, which leaves the ability of *F. philomiragia* to infect mice in question and untested (Mikalsen and Colquhoun, 2009; Cowley and Elkins, 2011).

Near-drowning victims are susceptible to numerous bacterial infections due to the direct inoculation of the bacteria into the lungs (Ender and Dolan, 1997; Relich et al., 2015). *F. philomiragia* infections have been reported in otherwise healthy individuals via direct lung exposure resulting from near-drowning experiences in brackish or salty water or immunocompromised individuals with contact to contaminated water or fish (Wenger et al., 1989; Ender and Dolan, 1997; Cora et al., 2010; Kreitmann et al., 2015). Despite differences in genomic sequences (88% homologous to *F. tularensis* subsp. *LVS* and 84% to *F. tularensis* subsp. *novicida* and *SchuS4*; Zeytun et al., 2012; Davenport et al., 2014; Johnson et al., 2015), slightly different plasmids (Le Pihive et al., 2009), and reports that *F. philomiragia* does not cause disease in mice (Mikalsen et al., 2009), some of these near drowning victims infected by *F. philomiragia* develop a severe pneumonic infection. This prompted further investigation on the similarity of *F. philomiragia* to *F. tularensis* subsp. *novicida* and subsp. *LVS*, strains related to virulent *F. tularensis* subsp. *SchuS4*, and whether it may be an opportunistic pathogens in humans. This comparison was achieved through the use of *in vitro* experiments using cell models involved in tularemia infections (macrophages, lungs, and liver) and *in vivo* animal infection models (*G. mellonella* and BALB/c mice).

## Materials and Methods

### Bacterial Strains

*Francisella tularensis* Live Vaccine Strain (*LVS*; ATCC 29684), *F. tularensis* subsp. *novicida* (ATCC 15482), and *F. philomiragia* (ATCC 25015) were obtained from the American Type Culture Collection (Manassas, VA, USA). All bacterial strains were streaked onto Chocolate II Agar (GC II Agar with Hemoglobin and IsoVitaleX^TM^, BD 221267) and single colonies were inoculated into Brain Heart Infusion (BHI pH 6.8) broth (TekNova, Hollister, CA, USA).

### Tissue Culture Cells

Murine macrophages, J774A.1 (ATCC TIB-67), human hepatocellular carcinoma cells, HepG2 (ATCC HB-8065), and human Type II alveolar epithelial cells, A549 (ATCC CCL-185), were cultured in Dulbecco’s Modified Eagle’s Medium (DMEM; Invitrogen #10566-016) supplemented with 10% fetal bovine serum (FBS) as per the manufacturer’s recommendations. Human U937 macrophage-like cells (ATCC CRL-1593.2), were cultured in RPMI 1640 media with 2 mM L-glutamine and 10% FBS as per the manufacturer’s recommendations (Lonza # 3163826). U937 cells were differentiated from monocytes to macrophages as instructed by manufacturer.

### Infection Protocol for A549, J774A.1, HepG2, and U937 Cells

Cells were infected at a multiplicity of infection (MOI) of approximately 500, as previously described (Han et al., 2008; Hegedus et al., 2008; Ahmad et al., 2010), with a 2-h preinfection and 1-h gentamicin pulse. Briefly, cells were seeded (10^5^/well) in a 48-well plate and allowed to attach overnight. After verifying successful cell attachment, culture media was gently removed and rinsed twice with culture media. *Francisella* strains were grown to mid-logarithmic phase, collected by centrifugation (10 min at 4000 × *g*, 4°C), washed three times with 1x phosphate buffered saline (PBS), and diluted in serum-free DMEM to a verified bacterial concentration (CFU/mL). Dilutions of bacteria were used to infect each cell line at MOI = 500. Sets of three wells were prepared for each condition (*n* = 3). Characteristically, *Francisella* infects host cells inefficiently, despite its infectivity via multiple routes in animals and humans. Therefore, the standard MOI of 500 CFU was used to infect cells with the *Francisella* strains in order to achieve infection of most of the cells (Lai et al., 2001; Lai and Sjostedt, 2003). Cells were then incubated with bacteria at 37°C, 5% CO_2_. After a 2-h incubation, well media was gently aspirated, washed twice with PBS, and treated with 50 μg/mL gentamicin in serum-free DMEM for 1 h to kill extracellular bacteria. Following the gentamicin pulse, cell media was gently aspirated, replaced with DMEM supplemented with 10% FBS and 5 μg/mL gentamicin, and allowed to incubate for 24 h at 37°C, 5% CO_2_. (Lai et al., 2001; Han et al., 2008; Ahmad et al., 2010) Cells were lysed and plated on Chocolate agar for CFU determination.

### Cytotoxicity Assay of Mammalian Tissue Cultured Cells Infected with *Francisella*

PrestoBlue Cell Viability Reagent (A-13261, Life Technologies, Carlsbad, CA, USA) was used according to the manufacturer’s protocol. This reagent functions by using the reducing environment of the cell’s cytosol to determine cell viability. The reagent contains a cell-permeable compound, which is blue in color. When added to viable cells, it encounters the reducing environment and modifies the reagent to become a red fluorescent, which can be detected by fluorescence or absorbance measurements. Briefly, reagent was added to infected cells 24 h post gentamicin-pulse at a 1:10 ratio. The reagent was incubated with cells at 37°C for 2 h. Fluorescence was measured at excitation and emission spectra of 560 and 590 nm, respectively. Three wells were used per condition (*n* = 3). Data was averaged and a no-cell well was subtracted as background. Data was then plotted using GraphPad Prism 5 (GraphPad Software Inc., San Diego, CA, USA) to reflect the cytotoxic effect of *Francisella* species on eukaryotic cell lines.

### EC_50_ Antimicrobial Assays

Peptides used in this study were custom synthesized by ChinaPeptides Company (Shanghai, China) and had purities of ≥95% based on chromatographic analysis of the purified peptides. Antimicrobial activity (EC_50_) assays of the antibiotic control levofloxacin, human cathelicidin LL-37, and murine cathelicidin mCRAMP were performed against *F. philomiragia*, *F. tularensis* subsp. *novicida, and F. tularensis* subsp. *LVS* as previously described (Amer et al., 2010). Briefly, 1 × 10^5^ CFU/well of *Francisella* species were grown in BHI (pH 6.8), added to a sterile 96-well plate and incubated with serial dilutions of peptide or antibiotic in 10 mM phosphate buffer for 3 h at 37°C. Dilutions were plated in triplicate on tryptic soy agar with 1% cysteine for 24 h; colonies were counted to determine survival (*n* = 3). This experiment was performed three independent times. Bacterial survival was calculated by a ratio of the number of colonies on each experimental plate to the average number of colonies on the control plates lacking peptide or antibiotic application. The EC_50_ was determined using GraphPad Prism 5 (GraphPad Software Inc., San Diego, CA, USA) to plot the percent survival versus log of peptide or antibiotic concentration (log μg/mL) and fitting data to a standard sigmoidal dose–response curve as previously described (Blower et al., 2015).

### Waxworm Infection

*Galleria mellonella* larvae (Vander horst Wholesale, St. Mary’s, OH, USA, 16 per group) were infected following previous reports (Aperis et al., 2007; Dean et al., 2011; McKenney et al., 2012). *G. mellonella* were infected by injecting 10 μL of bacteria into the hemocoel via a right proleg and incubated at 37°C. Each larva received bacterial concentrations of 1 × 10^6^, 5 × 10^5^, 1 × 10^5^, 5 × 10^4^, 1 × 10^4^, 5 × 10^3^, 1 × 10^3^, 5 × 10^2^, or 1 × 10^2^ CFU/mL with 16 larvae per group. Waxworms were examined once a day for death. Bacterial concentrations were verified via retrospective plating and counting of CFUs.

### Murine Infection

BALB/c mice (Harlan, Frederick, MD, USA, five per group) were infected intranasally with 20 μL of the following concentrations of bacteria: 1 × 10^6^, 5 × 10^5^, 1 × 10^5^, 5 × 10^4^, 1 × 10^4^, 5 × 10^3^, or 1 × 10^3^ CFU/20 μL. Mice were examined twice a day for signs of illness or death. Bacterial concentrations were verified via retrospective plating and counting of CFUs. Animal experiments were approved by and conducted in compliance with regulations of the Institutional Animal Care and Use Committee (Protocol # 0236) of George Mason University. All experiments were carried out in accordance with the National Research Council’s Guide for the Care and Use of Laboratory Animals (2011) and the Public Health Service Policy on Humane Care and Use of Laboratory Animals (2002).

### Statistical Analysis

Antimicrobial EC_50_ assays were performed in triplicate with *n* = 3 for each experiment, and representative experiments are shown. Standard deviations of the mean of each set are represented on each graph as error bars. Additionally the confidence interval (95%) is provided for EC_50_ determinations to demonstrate statistical overlap of data. Student’s *t-*test was performed and p values of *p* < 0.05 was considered statistically different.

The survival curves were performed with an *n* = 16 for *G. mellonella* and an *n* = 5 for BALB/c mice and were analyzed using the Mantel–Cox test, which is used to test the null hypothesis that survival curves are not different between groups. This test does not assume a normal distribution, allows for censored data, and is based off of the chi-squared test, which allows for a minimum of five samples.

## Results

During pulmonary tularemia infections, bacteria colonize the alveolar macrophages, the lungs, and the liver (Hall et al., 2007; Faron et al., 2015). *F. philomiragia* was evaluated to see if it infected cell lines representative of these systems *in vitro*: murine macrophage cells, J774A.1, human Type II alveolar epithelial cells, A549, and human hepatocyte-like cells, HepG2. These cell lines have been previously shown by us and others to be susceptible to infection by *F. tularensis* subsp. *novicida* and *LVS* (Qin and Mann, 2006; Han et al., 2008; Amer et al., 2010; Bradburne et al., 2013).

**FIGURE 1:**
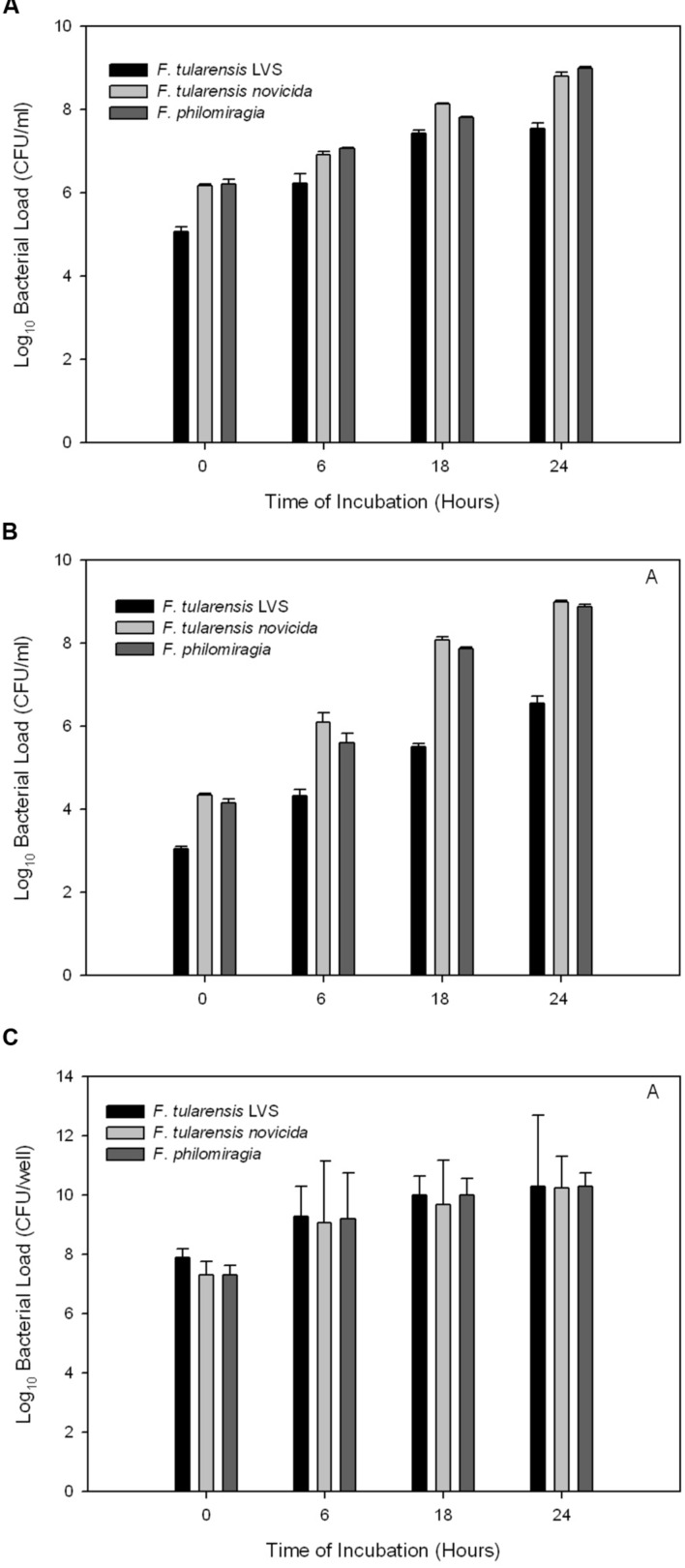
**Bacterial replication following infection of cultured mammalian cells.** *Francisella philomiragia, F. tularensis* subsp. *novicida, and F. tularensis* subsp. *LVS* infection of **(A)** murine macrophage J774A.1 cells, **(B)** human lung epithelial A549 cells, and **(C)** human hepatocytes HepG2 cells.

*Francisella tularensis* readily infects macrophages and proliferates within these cells (Anthony et al., 1991; Golovliov et al., 1997; Lai et al., 2001; Bolger et al., 2005). It is characteristic for *Francisella* replication to occur with little cytotoxicity until the cell becomes overburdened (at about 48 h post infection) and will experience cell death. *F. philomiragia* was found to be able to infect and proliferate in murine macrophages at 24 h to higher levels than what was seen for *F. tularensis* subsp. *novicida* and subsp. *LVS* (**Figure 1A**, *p* < 0.05). These results with the *in vitro* macrophage model suggest that *F. philomiragia* may be capable of infecting mammalian macrophages *in vivo*. Furthermore, these results suggest that *F. philomiragia* may be able to infect the alveolar macrophages in the lungs of near-drowning victims, which results in the clinical disease resembling tularemia that can aﬄict these patients.

Human alveolar epithelial cells are known to be infected by *Francisella* both *in vitro* and *in vivo* (Hall et al., 2007; Faron et al., 2015). Here, experiments utilizing A549 cells showed that *F. philomiragia* infects this cell type to a lesser extent than *F. tularensis* subsp. *novicida* but more than subsp. *LVS* (**Figure 1B**, *p* < 0.05). The infection of this cell type suggests another potential mechanism by which the near-drowning infections in human could occur by this organism due to the direct inoculation of the lung.

*Francisella* infection of and proliferation in hepatocytes has been observed in human tularemia patients and animal models (Conlan and North, 1992; Lamps et al., 2004; Rasmussen et al., 2006; Ray et al., 2010). The fully virulent *F. tularensis* subsp. *SchuS4* replicates well in cultured HepG2 cells (Qin and Mann, 2006). In these experiments, *F. tularensis* subsp. *LVS* infected human hepatocyte-like cells well, and replicated faster than *F. tularensis* subsp. *novicida* and *F. philomiragia*. However, by 24 h post infection, there were no differences between the bacterial burdens of the three *Francisella* species in HepG2 cells (**Figure 1C**, *p* > 0.05). This suggests that the *F. philomiragia* infections could potentially lead to liver damage, consistent with a tularemia infection.

*Francisella tularensis* is said to be a “stealth” pathogen, promoting its intracellular survival by not causing high cytotoxicity, among other mechanisms (Sjostedt, 2006; Jones et al., 2014). It was previously shown that infections of A549 cells by *F. tularensis* subsp. *LVS* (at 500 MOI) for 24 h did not cause significant cytotoxicity, although CFU increased significantly (Han et al., 2008; Bradburne et al., 2013). This high MOI of 500 is standard for *Francisella* infection protocols, as it is not taken up into non-phagocytic cells readily (Lai et al., 2001; Lai and Sjostedt, 2003; Telepnev et al., 2003). These studies were expanded to all three strains of *Francisella* investigated here (*F. tularensis* subsp. *LVS*, *F. tularensis* subsp. *novicida*, and *F. philomiragia*) and J774A.1, A549, and HepG2 cells.

**FIGURE 2:**
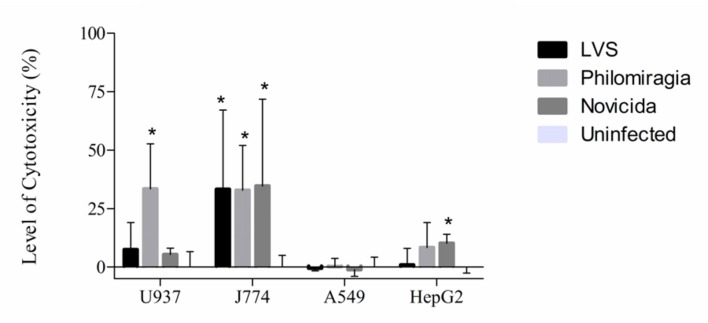
**Cytotoxicity of *Francisella* strains to cultured mammalian cells.** Cytotoxicity of *F. philomiragia* to cultured mammalian cells, compared to *F. tularensis* subsp. *LVS* and *F. tularensis* subsp. *novicida*.

This study confirmed that *F. tularensis* subsp. *LVS* is not significantly cytotoxic toward A549 cells and, furthermore, it was found that *F. tularensis* subsp. *novicida* and *F. philomiragia* also displayed little cytotoxicity in this cell line at 24 h (**Figure 2**). HepG2s were also minimally affected by cytotoxic effects of *F. tularensis* subsp. *LVS* and displayed only 10 and 8% cytotoxicity from *F. tularensis* subsp. *novicida* and *F. philomiragia* infections at 24 h, respectively. The murine macrophage cell line, J774A.1, demonstrated greater susceptibility to the cytotoxic effects of *Francisella*, with all strains demonstrating about 33% cytotoxicity at 24 h (*p* < 0.05) consistent with previous reports (Lai et al., 2001; Lai and Sjostedt, 2003). However, additional cytotoxicity studies showed that *F. philomiragia* is highly cytotoxic to the human macrophage-like cell line, U937, (33%, *p* < 0.05) while *F. tularensis* subsp. *LVS* and subsp. *novicida* showed only 7 and 5% cytotoxicity, respectively. The differences between the human and murine macrophage cell lines are not yet understood in regard to *Francisella* infections. Previously, differences in *Francisella* intracellular replication have been noted between rat and murine macrophages (Anthony et al., 1991), however, other causes for the cytotoxicity differences other than species of origin are possible. These findings are consistent with the intracellular replication lifestyle of other *Francisella* species (Sjostedt, 2006).

Here, *F. philomiragia* was found to infect and replicate in the same cell types and have the same general level of cytotoxicity in those cell types as *F. tularensis* subsp. *LVS* and subsp. *novicida*, with the exception of the U937 cells (*p* < 0.05). The susceptibility of *F. philomiragia* to two antimicrobial peptides, LL-37, a human cathelicidin, and mCRAMP, a murine cathelicidin, known to be expressed by host cells and have killing activity against *F. tularensis* subsp. *novicida* and subsp. *LVS* was examined (Amer et al., 2010). This is important because host defense against *Francisella* infection relies not only on antibody production, but also on the response of the innate immune system (Allen, 1962; Metzger et al., 2007; Kirimanjeswara et al., 2008).

**FIGURE 3:**
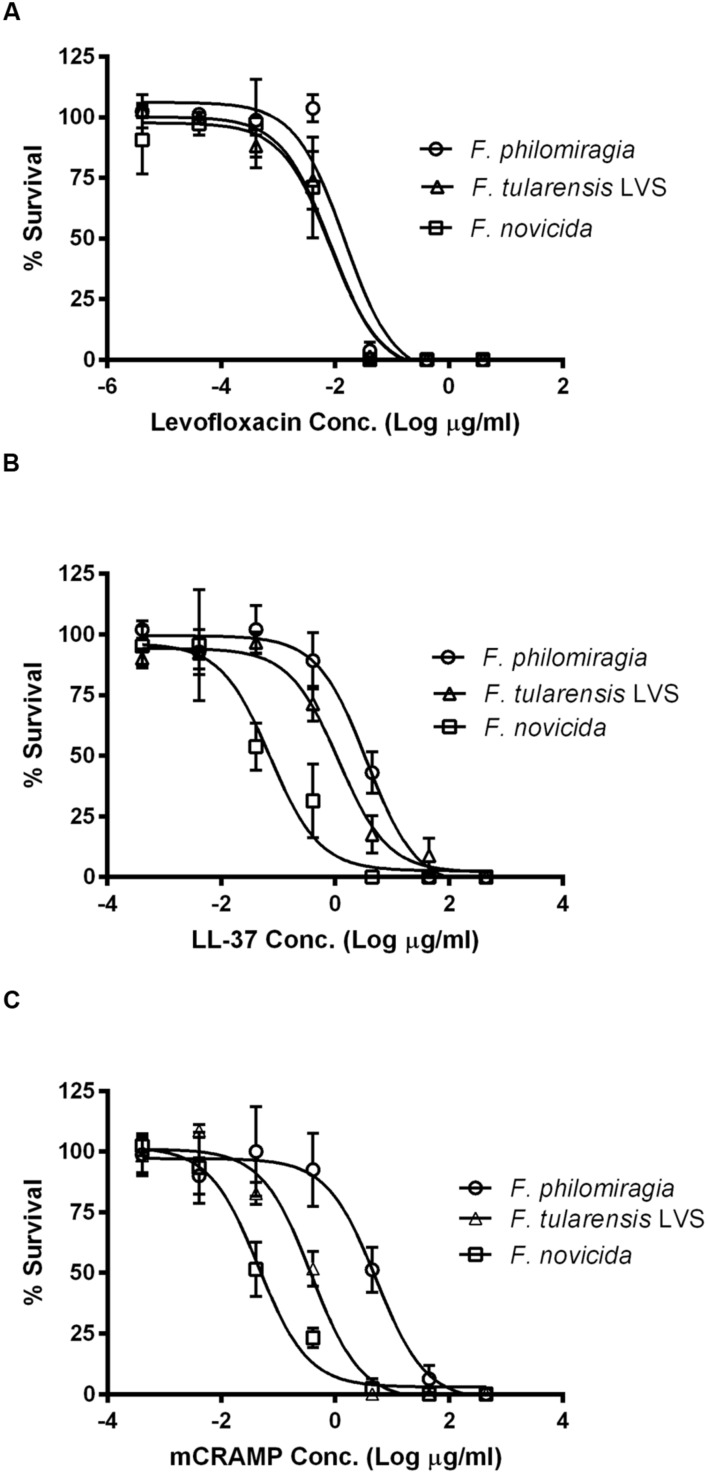
**Antimicrobial (EC_50_) assays for *Francisella* strains.** *F. philomiragia*, *F. tularensis* subsp. *novicida, and F. tularensis* subsp. *LVS* susceptibility against **(A)** levofloxacin and the cathelicidins **(B)** LL-37 and **(C)** mCRAMP.

*Francisella* species, including *F. philomiragia*, are known to be highly susceptible to levofloxacin under MIC conditions (Nelson et al., 2010; Georgi et al., 2012), thus it was used as a control for the EC_50_ antimicrobial assays. The EC_50_ for levofloxacin of *F. philomiragia* is 0.0146 μg/mL (14.6 ng/mL; **Figure 3A**) while the *F. tularensis* subsp. *LVS* EC_50_ is 0.00827 μg/mL (8.27 ng/mL) and the *F. tularensis* subsp. *novicida* EC_50_ is 0.00843 μg/mL (8.43 ng/mL; **Table 1**). These values are statistically the same within the 95% confidence intervals (*p* > 0.05) and are consistent with the MICs previously reported (Georgi et al., 2012).

**Table 1:** Summary of Antimicrobial (EC_50_) assays for *Francisella* strains.

		*Francisella tularensis* subsp. *LVS*	*Francisella tularensis* subsp. *novicida*	*Francisella philomiragia*
Levofloxacin	EC_50_ (μg/ml)	0.00827	0.00843	0.0146
	95% CI	(0.00524–0.0131)	(0.00413–0.0172)	(0.00696–0.0305)
mCRAMP	EC_50_ (μg/ml)	0.381	0.0453	5.27
	95% CI	(0.239–0.607)	(0.0284–0.0723)	(2.93–9.46)
LL-37	EC_50_ (μg/ml)	1.15	0.0724	3.61
	95% CI	(0.604–2.18)	(0.0331–0.158)	(2.36–5.53)

*EC_*50*_ values plus 95% confidence intervals for *F. philomiragia*, *F. tularensis* subsp. *novicida, and F. tularensis* subsp. *LVS* susceptibility against levofloxacin and the cathelicidins LL-37 and mCRAMP.*

The sensitivity of *F. philomiragia* to cationic antimicrobial peptides has not been well studied. This organism is highly resistant to colistin and polymyxin B, which are cationic cyclic peptide antibiotics (Petersen et al., 2009; Stephens et al., 2016). It was previously demonstrated that expression of the human cathelicidin LL-37 in A549 cells is strongly induced by *F. tularensis* subsp. *novicida* infection (Amer et al., 2010). This is of interest as *Francisella* bacteria replicate directly in the cytosol of the infected cells (Wehrly et al., 2009), and thus the bacteria may be able to be killed by expression of these innate immunity peptides by the aﬄicted cell.

The antimicrobial peptides, LL-37 and mCRAMP, were tested for their killing activity against *F. philomiragia* in 10 mM phosphate buffer, pH 7.2. The EC_50_ of LL-37 against *F. philomiragia* was determined to be 3.61 μg/mL (**Figure 3B**). In contrast to *Francisella* sensitivity to LL-37 in other species (EC_50_ of 1.15 and 0.0724 μg/mL in *F. tularensis* subsp. *LVS* and *novicida*; Amer et al., 2010; Flick-Smith et al., 2013), *F. philomiragia* is more resistant to this human cathelicidin peptide (*p* < 0.05). The EC_50_ of LL-37 against *F. tularensis* subsp. *novicida* found here is statistically similar to previously reported values due to overlapping 95% confidence interval values (Amer et al., 2010). Some small difference could also be due to the “host-adapted phenotype” growth conditions used to grow the bacteria for this study (BHI pH 6.8) compared to growth in Tryptic Soy Broth with Cysteine (TSB-C) media that was used previously (Amer et al., 2010). Growth in BHI (pH 6.8) is known to alter the surface carbohydrate and gene expression in a way that mimics the “host-adapted” phenotype of *Francisella* (Zarrella et al., 2011). In conclusion, *F. philomiragia* is more resistant to LL-37 than other *Francisella* species that were verified here (*F. tularensis* subsp. *LVS* EC_50_ = 1.15 (threefold), *p* = 0.011, and *F. tularensis* subsp. *novicida* EC_50_ = 0.0724 μg/mL (50-fold), *p* = 0.0011; Amer et al., 2010; Flick-Smith et al., 2013).

*Francisella* susceptibility to mCRAMP has not been previously reported. The EC_50_ of mCRAMP against *F. philomiragia* was determined to be 5.27 μg/mL (**Figure 3C**). No previous reports of *Francisella* susceptibility to mCRAMP were found; however, for this murine cathelicidin peptide, *F. philomiragia* is significantly more resistant (*p* < 0.05) than *F. tularensis* subsp. *novicida* (EC_50_ = 0.0453 μg/mL) or *F. tularensis* subsp. *LVS* (EC_50_ = 0.381 μg/mL). Here it was found that *F. philomiragia* is 14–116-fold more resistant to mCRAMP (EC_50_ = 5.27 μg/mL) than *F. tularensis* subsp. *LVS* (0.381 μg/mL, *p* = 0.007) and subsp. *novicida* (0.0453 μg/mL, *p* = 0.0051).

This increased resistance of *F. philomiragia* to cationic antimicrobial peptides could be due to differences in the LPS (Siddaramappa et al., 2012) or other surface properties of *F. philomiragia* compared to *F. tularensis* subsp. *novicida* or *LVS* perhaps due to differential expression of high-molecular weight carbohydrates in the “host-adapted” phenotype (Zarrella et al., 2011).

**FIGURE 4:**
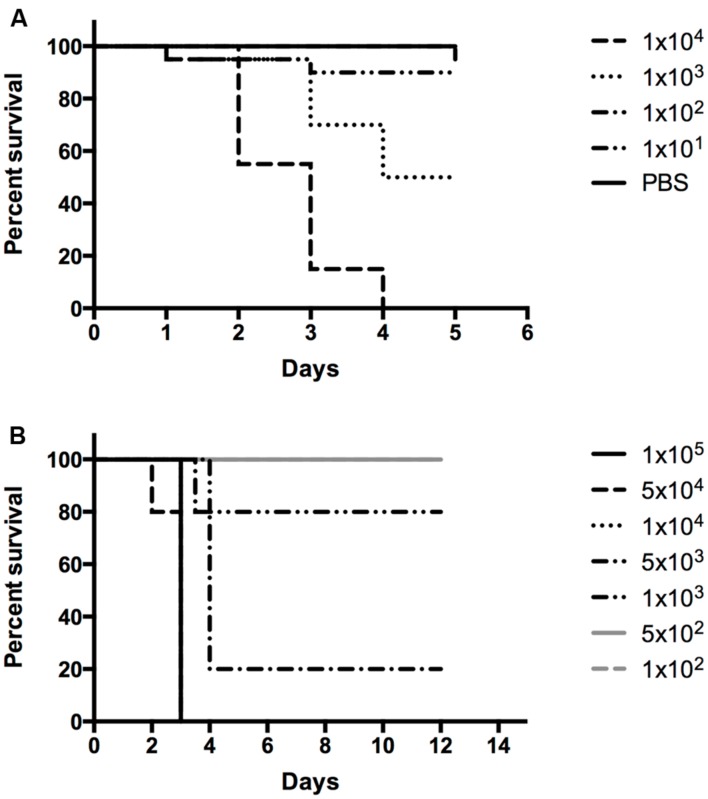
***In vivo* survival assays.** *F. philomiragia* survival curve in **(A)** *G. mellonella* and **(B)** BALB/c mice infected by the pulmonary route.

*Galleria mellonella* has been demonstrated to be a useful model for *Francisella* infection (Aperis et al., 2007; Ahmad et al., 2010; McKenney et al., 2012; Sprynski et al., 2014), thus a survival curve evaluating the mortality of *G. mellonella* during *F. philomiragia* infection was examined. As shown in **Figure 4A**, *F. philomiragia* injection is lethal to *G. mellonella* in a manner similar to *F. tularensis* subsp. *LVS* (Aperis et al., 2007; Ahmad et al., 2010). The LD_50_ of *F. philomiragia* in *G. mellonella* is ~1.8 × 10^3^ CFU/mL or ~18 CFU with an inoculation volume of 10 μL. For comparison, the LD_50_ of *F. tularensis* subsp. *novicida* in *G. mellonella* is ~1.2 × 10^2^ CFU/mL or ~1 CFU due to an inoculation volume of 10 μL (McKenney et al., 2012). The mean and median times to death for *F. philomiragia* are 2.79 and 3 days, respectively. Since *F. philomiragia* is able to infect *G. mellonella* similarly to other laboratory strains of *Francisella*, infection of BALB/c mice by the intranasal route of infection was tested for this organism.

BALB/c mice are a common experimental model for *Francisella* infections and they are susceptible to *Francisella* infection by the pulmonary route (intranasal or aerosol), among other routes; no studies have been reported for *F. philomiragia* infections of insect models, mice, rats, or marmosets (Chen et al., 2003; Conlan et al., 2003; Cowley and Elkins, 2011). To conform to these standards and expand the *in vivo* results obtained with *G. mellonella*, a survival curve evaluating the lethality of *F. philomiragia* in BALB/c mice when delivered via intranasal administration (mimicking near-drowning experiences) was examined. As shown in **Figure 4B**, intranasal *F. philomiragia* is lethal to BALB/c mice with an approximate LD_50_ of 3.45 × 10^3^ CFU. This is very comparable to the intranasal *F. tularensis* subsp. *LVS* LD_50_ (1 × 10^3^ CFU) in the same species of mice but is higher than the 100 CFU intranasal LD_50_ of *F. tularensis* subsp. *novicida* (Aperis et al., 2007).

## Discussion

Multiple reports of severe pneumonic infections of humans following near-drowning experiences (Hollis et al., 1989; Wenger et al., 1989) suggested that direct or large inoculation of *F. philomiragia* into the lung by this method is sufficient to allow for infection of normal, healthy human lungs, potentially via infection of the alveolar macrophages and/or lung epithelial cells. However, this organism is not generally regarded as a human pathogen and its ability to infect mammalian cells is generally uncharacterized. In addition, the highly related organism, *F. noatunensis*, was found to be unable to infect laboratory mice (Mikalsen et al., 2009). Thus, *F. philomiragia* was compared to *F. tularensis* subsp. *novicida* and subsp. *LVS* regarding its ability to infect human and murine cells was further studied.

*Francisella philomiragia* was shown to be capable of infecting a murine macrophage cell line, J774A.1, which are commonly used for *Francisella* studies, with statistically higher levels (*p* < 0.05) than more commonly studied strains of *Francisella* (Hegedus et al., 2008; Pechous et al., 2008; Ahmad et al., 2010). Similarly, *F. philomiragia* was also found to infect a human Type II alveolar epithelial cell line, A549, at statistically higher levels than *F. tularensis* subsp. *LVS*. This is the first demonstration of *F. philomiragia* infecting Type II alveolar epithelial cells and is a significant contribution to the understanding of the potential interactions of *F. philomiragia* within the human lung. These findings suggest a potential mechanism by which near-drowning in brackish water known to contain *F. philomiragia* (Ottem et al., 2007) could potentially lead to infection through interaction of the bacteria with Type II alveolar epithelial cells of the lung and/or alveolar macrophages (Gentry et al., 2007; Hall et al., 2007; Craven et al., 2008; Faron et al., 2015). Furthermore, these results suggest that aerosol exposure to *F. philomiragia* could potentially lead to pulmonary infections in humans if inhaled via an aerosol. Given the wide distribution of *F. philomiragia*, in particular its known presence in various bodies of water within the United States, this potential route of infection should be further investigated.

In addition, it was demonstrated that *F. philomiragia* infects HepG2 cells, a human hepatocyte-like cell line. This finding suggests that *F. philomiragia* may be able to replicate in the liver in infected near-drowning victims. The liver is one of the main organs infected by *F. tularensis* strains and liver failure following overwhelming organ infection is thought to be the primary cause of death in mice suffering from tularemia (Conlan and North, 1992). Patients suffering from *F. philomiragia* pneumonia should be closely observed for sequelae similar to those found in tularemia infections caused by *F. tularensis* species.

Cytotoxicity data after 24 h of infection show that *F. philomiragia* is similar to *F. tularensis* subsp. *novicida* and subsp. *LVS* in most of the studied cell lines. Little cytotoxicity was seen in A549 cells (~0%, similar to other species) and HepG2 cells (8%, more than subsp. *LVS* but similar to subsp. *novicida*), and moderate cytotoxicity in J774A.1 cells (32%, similar to other species). However, the U937 human macrophage-like cell line only showed high cytotoxicity (33%) from *F. philomiragia* and not the other *Francisella* strains studied. This observation will be the subject of future investigation to understand the difference in U937 susceptibility.

Susceptibility testing using the antimicrobial peptides LL-37, a human cathelicidin, and mCRAMP, a murine cathelicidin, showed that these peptides were highly active *in vitro* against *F. philomiragia*. Despite being active in killing the bacteria *in vitro*, this antimicrobial peptide host defense mechanism is clearly insufficient to control *F. philomiragia* infections in infected cells or *in vivo.*

*Francisella philomiragia* was found to be lethal for both *in vivo* models tested: *G. mellonella* and BALB/c mice. *G. mellonella* has been demonstrated to be a useful *in vivo* model for *Francisella* infection (Aperis et al., 2007; Ahmad et al., 2010; McKenney et al., 2012; Sprynski et al., 2014), thus the survival of *G. mellonella* during *F. philomiragia* infection was examined. In *G. mellonella*, *F. philomiragia* was shown to be fatal in concentrations similar to *F. tularensis* subsp. *LVS*, with an LD_50_ of 18 CFU. This similarity supports the ability of *G. mellonella* to be used as an effective model for *Francisella* infection but also suggests that *F. philomiragia* is capable of infecting a range of hosts similar to other *Francisella* strains.

*Francisella philomiragia* is not generally regarded as a pathogen of humans or animals but is considered an environmental species of the genus (Anda et al., 2001; Tarnvik et al., 2004; Verhoeven et al., 2010). In some cases, *F. philomiragia* infections in near-drowning victims individuals are observed (Wenger et al., 1989; Ender and Dolan, 1997). An intranasal infection of mice by *F. philomiragia* was used to mimic lung exposure seen in drowning victims and test the susceptibility BALB/c mice to this organism. *F. philomiragia* was shown to be fatal in BALB/c mice by intranasal-delivered inoculum concentrations similar to *F. tularensis* subsp. *LVS*, with an LD_50_ of 3.45 × 10^3^ CFU; however, this is significantly higher than the 10 CFU LD_50_ seen with *F. tularensis* subsp. *novicida*. Thus, contrary to the result for *F. noatunensis* (Mikalsen et al., 2009), *F. philomiragia* is able to infect laboratory mice. These results call for further studies to determine the full host range of *F. philomiragia*.

## Conclusion

These studies show that *F. philomiragia* results in similar *in vitro* and *in vivo* infections to the *F. tularensis* subspecies *novicida* and *LVS* for the evaluated strains. It was demonstrated for the first time that there is potential for significant and robust *F. philomiragia* infection in macrophages, lung, and liver cells. *F. philomiragia* infection of human alveolar epithelial cells and macrophages suggests a mechanism for infection in the lungs of near-drowning patients. The high level of *F. philomiragia* intracellular replication in all three cell types suggests that *F. philomiragia* follows an infection course similar to tularemia caused by *F. tularensis* subspecies. It was previously demonstrated that infections of *G. mellonella* and pulmonary infections of BALB/c mice were fatal with similar LD_50_s to *F. tularensis* subsp. *LVS*. The results of these *in vitro* and *in vivo* experiments confirm earlier suggestions that *F. philomiragia* may be an emerging opportunistic human pathogen (Mailman and Schmidt, 2005; Sjodin et al., 2012) and that cellular and animal models of *Francisella* infection could also be used to study *F. philomiragia*. It would be of interest to evaluate all the available *F. philomiragia* strains for their ability to infect the various tissue culture and murine models.

It was found that *F. philomiragia* is comparable to the other Biosafety level 2 strains of *Francisella* in many respects but unusual in its effect on human U937 cells. This finding will open some interesting new avenues of research regarding pathogenesis and virulence of *F. philomiragia*. In addition, this work also positions *F. philomiragia* as another important organism in the field of *Francisella* research, especially for researchers interested in questions of microbial ecology or environmental persistence of members of the genus *Francisella*.

## Author Contributions

All authors listed have made substantial, direct and intellectual contribution to the work, and approved it for publication. MLV conceived the study; MLV and CNP wrote the manuscript; CNP, SLP, RJB, SA, and MM contributed experimental data and contributed to the manuscript.

## Conflict of Interest Statement

The authors declare that the research was conducted in the absence of any commercial or financial relationships that could be construed as a potential conflict of interest.

## References

[B1] Ahmad S. Hunter L. Qin A. Mann B. J. van Hoek M. L. (2010). Azithromycin effectiveness against intracellular infections of Francisella. *BMC Microbiol.* 10:123 10.1186/1471-2180-10-123 PMC2881020 20416090

[B2] Allen W. P. (1962). Immunity against tularemia: passive protection of mice by transfer of immune tissues. *J. Exp. Med.* 115 411–420. 10.1084/jem.115.2.411 13860583 PMC2137492

[B3] Amer L. S. Bishop B. M. van Hoek M. L. (2010). Antimicrobial and antibiofilm activity of cathelicidins and short, synthetic peptides against *Francisella*. *Biochem. Biophys. Res. Commun.* 396 246–251. 10.1016/j.bbrc.2010.04.073 20399752

[B4] Anda P. Segura del Pozo J. Diaz Garcia J. M. Escudero R. Garcia Pena F. J. Lopez Velasco M. C. (2001). Waterborne outbreak of tularemia associated with crayfish fishing. *Emerg. Infect. Dis.* 7(3 Suppl.), 575–582. 10.3201/eid0703.010340 11485678 PMC2631832

[B5] Anthony L. D. Burke R. D. Nano F. E. (1991). Growth of *Francisella* spp. in rodent macrophages. *Infect. Immun.* 59 3291–3296. 1879943 10.1128/iai.59.9.3291-3296.1991 PMC258167

[B6] Aperis G. Fuchs B. B. Anderson C. A. Warner J. E. Calderwood S. B. Mylonakis E. (2007). *Galleria mellonella* as a model host to study infection by the *Francisella tularensis* live vaccine strain. *Microbes Infect.* 9 729–734. 10.1016/j.micinf.2007.02.016 17400503 PMC1974785

[B7] Berrada Z. L. Telford S. R. III (2010). Diversity of Francisella species in environmental samples from Martha’s Vineyard. Massachusetts. *Microb. Ecol.* 59 277–283. 10.1007/s00248-009-9568-y 19669828 PMC2836248

[B8] Blower R. J. Barksdale S. M. van Hoek M. L. (2015). Snake cathelicidin NA-CATH and smaller helical antimicrobial peptides are effective against *Burkholderia thailandensis*. *PLoS Negl. Trop Dis.* 9:e0003862 10.1371/journal.pntd.0003862 PMC4510350 26196513

[B9] Bolger C. E. Forestal C. A. Italo J. K. Benach J. L. Furie M. B. (2005). The live vaccine strain of *Francisella tularensis* replicates in human and murine macrophages but induces only the human cells to secrete proinflammatory cytokines. *J. Leukoc. Biol.* 77 893–897. 10.1189/jlb.1104637 15758077

[B10] Bradburne C. E. Verhoeven A. B. Manyam G. C. Chaudhry S. A. Chang E. L. Thach D. C. (2013). Temporal transcriptional response during infection of type II alveolar epithelial cells with *Francisella tularensis* live vaccine strain (LVS) supports a general host suppression and bacterial uptake by macropinocytosis. *J. Biol. Chem.* 288 10780–10791. 10.1074/jbc.M112.362178 23322778 PMC3624459

[B11] Chen W. Shen H. Webb A. KuoLee R. Conlan J. W. (2003). Tularemia in BALB/c and C57BL/6 mice vaccinated with *Francisella tularensis* LVS and challenged intradermally, or by aerosol with virulent isolates of the pathogen: protection varies depending on pathogen virulence, route of exposure, and host genetic background. *Vaccine* 21 3690–3700. 12922099 10.1016/s0264-410x(03)00386-4

[B12] Conlan J. W. Chen W. Shen H. Webb A. KuoLee R. (2003). Experimental tularemia in mice challenged by aerosol or intradermally with virulent strains of *Francisella tularensis*: bacteriologic and histopathologic studies. *Microb. Pathog.* 34 239–248. 10.1016/S0882-4010(03)00046-9 12732472

[B13] Conlan J. W. North R. J. (1992). Early pathogenesis of infection in the liver with the facultative intracellular bacteria *Listeria monocytogenes*, *Francisella tularensis*, and *Salmonella typhimurium* involves lysis of infected hepatocytes by leukocytes. *Infect. Immun.* 60 5164–5171. 1452350 10.1128/iai.60.12.5164-5171.1992 PMC258293

[B14] Cora M. C. Neel J. A. Tarigo J. Post K. Barnes J. (2010). Francisella philomiragia septicemia in a dog. *J. Vet. Intern. Med.* 24 969–972. 10.1111/j.1939-1676.2010.0545.x 20649752

[B15] Cowley S. C. Elkins K. L. (2011). Immunity to *Francisella*. *Front. Microbiol.* 2:26 10.3389/fmicb.2011.00026 PMC3109299 21687418

[B16] Craven R. R. Hall J. D. Fuller J. R. Taft-Benz S. Kawula T. H. (2008). *Francisella tularensis* invasion of lung epithelial cells. *Infect. Immun.* 76 2833–2842. 10.1128/IAI.00043-08 18426871 PMC2446690

[B17] Dai S. Mohapatra N. P. Schlesinger L. S. Gunn J. S. (2010). Regulation of francisella tularensis virulence. *Front. Microbiol.* 1:144 10.3389/fmicb.2010.00144 PMC3109300 21687801

[B18] Davenport K. W. Daligault H. E. Minogue T. D. Bishop-Lilly K. A. Broomall S. M. Bruce D. C. (2014). Whole-genome sequences of nine francisella isolates. *Genome Announc* 2:5 10.1128/genomeA.00941-14 PMC4175199 25291764

[B19] Dean S. N. Bishop B. M. van Hoek M. L. (2011). Susceptibility of *Pseudomonas aeruginosa* Biofilm to Alpha-Helical Peptides: D-enantiomer of LL-37. *Front. Microbiol.* 2:128 10.3389/fmicb.2011.00128 PMC3131519 21772832

[B20] Ender P. T. Dolan M. J. (1997). Pneumonia associated with near-drowning. *Clin. Infect. Dis.* 25 896–907. 10.1086/515532 9356805

[B21] Faron M. Fletcher J. R. Rasmussen J. A. Apicella M. A. Jones B. (2015). Interactions of Francisella tularensis with alveolar type II epithelial cells and the murine respiratory epithelium. *PLoS ONE* 10:e0127458 10.1371/journal.pone.0127458 PMC4444194 26010977

[B22] Flick-Smith H. C. Fox M. A. Hamblin K. A. Richards M. I. Jenner D. C. Laws T. R. (2013). Assessment of antimicrobial peptide LL-37 as a post-exposure therapy to protect against respiratory tularemia in mice. *Peptides* 43 96–101. 10.1016/j.peptides.2013.02.024 23500517

[B23] Gentry M. Taormina J. Pyles R. B. Yeager L. Kirtley M. Popov V. L. (2007). Role of primary human alveolar epithelial cells in host defense against *Francisella tularensis* infection. *Infect. Immun.* 75 3969–3978. 10.1128/IAI.00157-07 17502386 PMC1951971

[B24] Georgi E. Schacht E. Scholz H. C. Splettstoesser W. D. (2012). Standardized broth microdilution antimicrobial susceptibility testing of *Francisella tularensis* subsp. holarctica strains from Europe and rare *Francisella* species. *J. Antimicrob. Chemother.* 67 2429–2433. 10.1093/jac/dks238 22763567

[B25] Golovliov I. Ericsson M. Sandstrom G. Tarnvik A. Sjostedt A. (1997). Identification of proteins of *Francisella tularensis* induced during growth in macrophages and cloning of the gene encoding a prominently induced 23-kilodalton protein. *Infect. Immun.* 65 2183–2189. 9169749 10.1128/iai.65.6.2183-2189.1997 PMC175301

[B26] Hall J. D. Craven R. R. Fuller J. R. Pickles R. J. Kawula T. H. (2007). *Francisella tularensis* replicates within alveolar type II epithelial cells in vitro and in vivo following inhalation. *Infect. Immun.* 75 1034–1039. 10.1128/IAI.01254-06 17088343 PMC1828526

[B27] Han S. Bishop B. M. van Hoek M. L. (2008). Antimicrobial activity of human beta-defensins and induction by *Francisella.* *Biochem. Biophys. Res. Commun.* 371 670–674. 10.1016/j.bbrc.2008.04.092 18452706

[B28] Hegedus C. M. Skibola C. F. Warner M. Skibola D. R. Alexander D. Lim S. (2008). Decreased urinary beta-defensin-1 expression as a biomarker of response to arsenic. *Toxicol. Sci.* 106 74–82. 10.1093/toxsci/kfn104 18511430 PMC2563143

[B29] Hollis D. G. Weaver R. E. Steigerwalt A. G. Wenger J. D. Moss C. W. Brenner D. J. (1989). *Francisella philomiragia* comb. nov. (formerly *Yersinia philomiragia*) and *Francisella tularensis* biogroup novicida (formerly *Francisella novicida*) associated with human disease. *J. Clin. Microbiol.* 27 1601–1608. 2671019 10.1128/jcm.27.7.1601-1608.1989 PMC267622

[B30] Johnson S. L. Daligault H. E. Davenport K. W. Coyne S. R. Frey K. G. Koroleva G. I. (2015). Genome sequencing of 18 francisella strains to aid in assay development and testing. *Genome Announc* 3 e00147 10.1128/genomeA.00147-15 PMC4417685 25931589

[B31] Jones B. D. Faron M. Rasmussen J. A. Fletcher J. R. (2014). Uncovering the components of the *Francisella tularensis* virulence stealth strategy. *Front. Cell Infect. Microbiol.* 4:32 10.3389/fcimb.2014.00032 PMC3945745 24639953

[B32] Kay W. Petersen B. O. Duus J. O. Perry M. B. Vinogradov E. (2006). Characterization of the lipopolysaccharide and beta-glucan of the fish pathogen *Francisella victoria*. *FEBS J.* 273 3002–3013. 10.1111/j.1742-4658.2006.05311.x 16759227

[B33] Kirimanjeswara G. S. Olmos S. Bakshi C. S. Metzger D. W. (2008). Humoral and cell-mediated immunity to the intracellular pathogen *Francisella tularensis*. *Immunol. Rev.* 225 244–255. 10.1111/j.1600-065X.2008.00689.x 18837786 PMC4871322

[B34] Kreitmann L. Terriou L. Launay D. Caspar Y. Courcol R. Maurin M. (2015). Disseminated infection caused by *Francisella philomiragia*. France, 2014. *Emerg. Infect. Dis.* 21 2260–2261. 10.3201/eid2112.150615 26583375 PMC4672438

[B35] Lai X. H. Golovliov I. Sjostedt A. (2001). Francisella tularensis induces cytopathogenicity and apoptosis in murine macrophages via a mechanism that requires intracellular bacterial multiplication. *Infect. Immun.* 69 4691–4694. 10.1128/IAI.69.7.4691-4694.2001 11402018 PMC98551

[B36] Lai X. H. Sjostedt A. (2003). Delineation of the molecular mechanisms of *Francisella tularensis-*induced apoptosis in murine macrophages. *Infect. Immun.* 71 4642–4646. 10.1128/IAI.71.8.4642-4646.2003 12874344 PMC165996

[B37] Lamps L. W. Havens J. M. Sjostedt A. Page D. L. Scott M. A. (2004). Histologic and molecular diagnosis of tularemia: a potential bioterrorism agent endemic to North America. *Mod. Pathol.* 17 489–495. 10.1038/modpathol.3800087 15001997

[B38] Le Pihive E. Blaha D. Chenavas S. Thibault F. Vidal D. Valade E. (2009). Description of two new plasmids isolated from *Francisella philomiragia* strains and construction of shuttle vectors for the study of *Francisella tularensis*. *Plasmid* 62 147–157. 10.1016/j.plasmid.2009.07.001 19615403

[B39] Mailman T. L. Schmidt M. H. (2005). Francisella philomiragia adenitis and pulmonary nodules in a child with chronic granulomatous disease. *Can. J. Infect. Dis. Med. Microbiol.* 16 245–248. 18159552 10.1155/2005/486417 PMC2095034

[B40] Mauel M. J. Soto E. Moralis J. A. Hawke J. (2007). A piscirickettsiosis-like syndrome in cultured Nile tilapia in Latin America with *Francisella* spp. as the pathogenic agent. *J. Aquat. Anim. Health* 19 27–34. 10.1577/H06-025.1 18236629

[B41] McKenney E. S. Sargent M. Khan H. Uh E. Jackson E. R. San Jose G. (2012). Lipophilic prodrugs of FR900098 are antimicrobial against *Francisella novicida* in vivo and in vitro and show GlpT independent efficacy. *PLoS ONE* 7:e38167 10.1371/journal.pone.0038167 PMC3471904 23077474

[B42] Metzger D. W. Bakshi C. S. Kirimanjeswara G. (2007). Mucosal immunopathogenesis of *Francisella tularensis*. *Ann. N. Y. Acad. Sci.* 1105 266–283. 10.1196/annals.1409.007 17395728

[B43] Mikalsen J. Colquhoun D. J. (2009). *Francisella asiatica* sp. nov. isolated from farmed tilapia (Oreochromis sp.) and elevation of *Francisella philomiragia* subsp. noatunensis to species rank as *Francisella noatunensis* comb. nov., sp. nov. *Int. J. Syst. Evol. Microbiol.* 10.1099/ijs.0.002139-0 [Epub ahead of print]. 19783606

[B44] Mikalsen J. Olsen A. B. Rudra H. Moldal T. Lund H. Djonne B. (2009). Virulence and pathogenicity of *Francisella philomiragia* subsp. noatunensis for Atlantic cod, Gadus morhua L., and laboratory mice. *J. Fish Dis.* 32 377–381. 10.1111/j.1365-2761.2008.00987.x 19335614

[B45] Nano F. E. Schmerk C. (2007). The Francisella pathogenicity island. *Ann. N. Y. Acad. Sci.* 1105 122–137. 10.1196/annals.1409.000 17395722

[B46] Nelson M. Lever M. S. Dean R. E. Pearce P. C. Stevens D. J. Simpson A. J. (2010). Bioavailability and efficacy of levofloxacin against *Francisella tularensis* in the common marmoset (*Callithrix jacchus*). *Antimicrob. Agents Chemother.* 54 3922–3926. 10.1128/AAC.00390-10 20625157 PMC2934983

[B47] Ostland V. E. Stannard J. A. Creek J. J. Hedrick R. P. Ferguson H. W. Carlberg J. M. (2006). Aquatic Francisella-like bacterium associated with mortality of intensively cultured hybrid striped bass *Morone chrysops* x *M. saxatilis*. *Dis. Aquat. Organ.* 72 135–145. 10.3354/dao072135 17140136

[B48] Ottem K. F. Nylund A. Karlsbakk E. Friis-Moller A. Krossoy B. Knappskog D. (2007). New species in the genus Francisella (Gammaproteobacteria; Francisellaceae); *Francisella piscicida* sp. nov. isolated from cod (Gadus morhua). *Arch. Microbiol.* 188 547–550. 10.1007/s00203-007-0274-1 17619856

[B49] Pechous R. D. McCarthy T. R. Mohapatra N. P. Soni S. Penoske R. M. Salzman N. H. (2008). A *Francisella tularensis* Schu S4 purine auxotroph is highly attenuated in mice but offers limited protection against homologous intranasal challenge. *PLoS ONE* 3:e2487 10.1371/journal.pone.0002487 PMC2429968 18575611

[B50] Petersen J. M. Carlson J. Yockey B. Pillai S. Kuske C. Garbalena G. (2009). Direct isolation of *Francisella* spp. from environmental samples. *Lett. Appl. Microbiol.* 48 663–667. 10.1111/j.1472-765X.2009.02589.x 19413814

[B51] Qin A. Mann B. J. (2006). Identification of transposon insertion mutants of *Francisella tularensis* tularensis strain Schu S4 deficient in intracellular replication in the hepatic cell line HepG2. *BMC Microbiol.* 6:69 10.1186/1471-2180-6-69 PMC1557513 16879747

[B52] Rasmussen J. W. Cello J. Gil H. Forestal C. A. Furie M. B. Thanassi D. G. (2006). Mac-1+ cells are the predominant subset in the early hepatic lesions of mice infected with *Francisella tularensis*. *Infect. Immun.* 74 6590–6598. 10.1128/IAI.00868-06 17000726 PMC1698106

[B53] Ray H. J. Chu P. Wu T. H. Lyons C. R. Murthy A. K. Guentzel M. N. (2010). The Fischer 344 rat reflects human susceptibility to francisella pulmonary challenge and provides a new platform for virulence and protection studies. *PLoS ONE* 5:e9952 10.1371/journal.pone.0009952 PMC2848594 20376351

[B54] Relich R. F. Humphries R. M. Mattison H. R. Miles J. E. Simpson E. R. Corbett I. J. (2015). *Francisella philomiragia* Bacteremia in a patient with acute respiratory insufficiency and acute-on-chronic kidney disease: a case report and review of the literature. *J. Clin. Microbiol.* 53 3947–3950. 10.1128/JCM.01762-15 26400786 PMC4652090

[B55] Siddaramappa S. Challacombe J. F. Petersen J. M. Pillai S. Kuske C. R. (2012). Genetic diversity within the genus Francisella as revealed by comparative analyses of the genomes of two North American isolates from environmental sources. *BMC Genomics* 13:422 10.1186/1471-2164-13-422 PMC3479022 22920915

[B56] Sjodin A. Svensson K. Ohrman C. Ahlinder J. Lindgren P. Duodu S. (2012). Genome characterisation of the genus *Francisella* reveals insight into similar evolutionary paths in pathogens of mammals and fish. *BMC Genomics* 13:268 10.1186/1471-2164-13-268 PMC3485624 22727144

[B57] Sjostedt A. (2006). Intracellular survival mechanisms of *Francisella tularensis*, a stealth pathogen. *Microbes Infect.* 8 561–567. 10.1016/j.micinf.2005.08.001 16239121

[B58] Sjostedt A. (2007). Tularemia: history, epidemiology, pathogen physiology, and clinical manifestations. *Ann. N. Y. Acad. Sci.* 1105 1–29. 10.1196/annals.1409.009 17395726

[B59] Sprynski N. Valade E. Neulat-Ripoll F. (2014). Galleria mellonella as an infection model for select agents. *Methods Mol. Biol.* 1197 3–9. 10.1007/978-1-4939-1261-2_1 25172272

[B60] Stephens M. D. Hubble V. B. Ernst R. K. van Hoek M. L. Melander R. J. Cavanagh J. (2016). Potentiation of *Francisella* resistance to conventional antibiotics through small molecule adjuvants. *Med. Chem. Commun. Adv.* 7 128–131. 10.1039/C5MD00353A PMC4749165 26877862

[B61] Tarnvik A. Priebe H. S. Grunow R. (2004). Tularaemia in Europe: an epidemiological overview. *Scand. J. Infect. Dis.* 36 350–355. 10.1080/00365540410020442 15287379

[B62] Telepnev M. Golovliov I. Grundstrom T. Tarnvik A. Sjostedt A. (2003). *Francisella tularensis* inhibits Toll-like receptor-mediated activation of intracellular signalling and secretion of TNF-alpha and IL-1 from murine macrophages. *Cell Microbiol.* 5 41–51. 10.1046/j.1462-5822.2003.00251.x 12542469

[B63] Verhoeven A. B. Durham-Colleran M. W. Pierson T. Boswell W. T. Van Hoek M. L. (2010). *Francisella philomiragia* biofilm formation and interaction with the aquatic protist *Acanthamoeba castellanii*. *Biol. Bull.* 219 178–188. 20972262 10.1086/BBLv219n2p178

[B64] Wehrly T. D. Chong A. Virtaneva K. Sturdevant D. E. Child R. Edwards J. A. (2009). Intracellular biology and virulence determinants of *Francisella tularensis* revealed by transcriptional profiling inside macrophages. *Cell Microbiol.* 11 1128–1150. 10.1111/j.1462-5822.2009.01316.x 19388904 PMC2746821

[B65] Wenger J. D. Hollis D. G. Weaver R. E. Baker C. N. Brown G. R. Brenner D. J. (1989). Infection caused by *Francisella philomiragia* (formerly *Yersinia philomiragia*). A newly recognized human pathogen. *Ann. Intern. Med.* 110 888–892. 10.7326/0003-4819-110-11-888 2541646

[B66] Whipp M. J. Davis J. M. Lum G. de Boer J. Zhou Y. Bearden S. W. (2003). Characterization of a novicida-like subspecies of *Francisella tularensis* isolated in Australia. *J. Med. Microbiol.* 52(Pt 9), 839–842. 10.1099/jmm.0.05245-0 12909664

[B67] Whitehouse C. A. Kesterson K. E. Duncan D. D. Eshoo M. W. Wolcott M. (2012). Identification and characterization of *Francisella* species from natural warm springs in Utah, USA. *Lett. Appl. Microbiol.* 54 313–324. 10.1111/j.1472-765X.2012.03214.x 22283482

[B68] Zarrella T. M. Singh A. Bitsaktsis C. Rahman T. Sahay B. Feustel P. J. (2011). Host-adaptation of *Francisella tularensis* alters the bacterium’s surface-carbohydrates to hinder effectors of innate and adaptive immunity. *PLoS ONE* 6:e22335 10.1371/journal.pone.0022335 PMC3142145 21799828

[B69] Zeytun A. Malfatti S. A. Vergez L. M. Shin M. Garcia E. Chain P. S. (2012). Complete genome sequence of *Francisella philomiragia* ATCC 25017. *J. Bacteriol.* 194:3266 10.1128/JB.00413-12 PMC3370849 22628499

